# Cross-Cultural Validation of the Barcelona Immigration Stress Scale

**DOI:** 10.1007/s10903-023-01520-2

**Published:** 2023-07-17

**Authors:** Francisco José Eiroa-Orosa, Stella Evangelidou, Adil Qureshi, Francisco Collazos

**Affiliations:** 1 Section of Personality, Assessment and Psychological Treatment, Department of Clinical Psychology and Psychobiology, Faculty of Psychology, Institute of Neurosciences, University of Barcelona, Passeig Vall d’Hebron, 171, 08035 Barcelona, Catalonia Spain; 2 Barcelona Institute for Global Health (ISGlobal), Barcelona, Catalonia Spain; 3 Department of Psychiatry, University Hospital Vall d’Hebron, CIBERSAM, Autonomous University of Barcelona, Barcelona, Catalonia Spain

**Keywords:** Acculturative stress, discrimination, homesickness, immigration stress, measurement, mental health, psychometrics, psychosocial

## Abstract

The concept of acculturative stress has been the subject of debate, resulting in contradictory arguments regarding its measurement. Many of the existing acculturative stress instruments have been developed for use with specific migrant groups. The objective of the current study is to revisit the psychometric properties of the Barcelona Immigration Stress Scale, an instrument aimed at measuring stress levels associated with the migration trajectory in any migrant group. Using a sample of 915 migrants who consulted in primary care, we carried out principal component and confirmatory factor analyses as well as item response theory analyses applied both to the entire sample and to different migratory groups separately. The new reduced version of the scale has very good psychometric properties both in general and applied to the Latino migratory group, the largest in the area where the study was carried out. Results were acceptable among sub-Saharan, North African, and Asian groups but not among Eastern Europeans. These results are discussed through the specificities of each migratory group and the limitations of psychometrics to capture the complexity of immigration-related and acculturative stress.

**Supplementary Information:**

The online version contains supplementary material available at 10.1007/s10903-023-01520-2.

## Introduction

### Stress, Migration and Mental Health

Stress refers to any event in which either the demands of the environment or internal pressures, or both, exceed the adaptive resources of the individual [1]. In recent decades, stress related to migration and intercultural contact has gained significant research attention as a way to understand the connection between migration and mental health. Migratory processes have become an important topic in clinical research due to the potential stressors involved and their repercussion on psychosocial stability. According to Bhugra [2] migrants often encounter cultural differences that necessitate adaptation, identity restructuring, identification of losses, and preparation for potential stressors associated with migration. If the crisis is not effectively resolved, this process can lead to distress that may intensify and increase the likelihood of a person suffering from chronic stress. Consequently, Patino and Kirchner [3] suggest that the host society must develop efficient tools to prevent mental health problems linked to migration.

## Immigration-Related Acculturation and Acculturative Stress

The classical definition of acculturation refers to the process of cultural change that migrating individuals and groups experience when they come into contact with individuals from the receiving culture [4]. Based on the bidirectional model of acculturation, this change describes acculturation in terms of two orientations: one’s relation to a home culture (culture of origin) and one´s relation to a host culture (a new, second culture) [5]. Schwartz and Unger [6] have exposed the difficulties in the clear and precise conceptualizations of acculturation as well as in the definition, operationalization and measurement of the construct in host countries.

The concept of acculturative stress has been debated and criticized by both clinicians and researchers concluding to contradictory arguments. Rudmin [7] has called for the dismissal of the acculturative stress construct in psychological research, arguing that it is illogical to create a stress-based construct based on a process that is not inherently stressful. This argument opposes Bhugra’s [2] earlier statement about the relationship between mental distress and migration. There are also inconsistencies in defining the domains that acculturative stress may encompass, with little emphasis on the contextual and multifaceted nature of acculturation processes [8].

Some researchers tend to stay “true” to the acculturative aspect of the construct; whereas others would appear to include stressors confronted by migrants and/or culturally different people that are not directly related to intercultural contact, a requisite for “true” acculturative stress. The inclusion of “discrimination” as a factor in the Acculturative Stress in the International Students Scale [9] or the Barcelona Immigration Stress Scale [10] exemplifies this issue. However, the factor is explicitly rejected in the Multidimensional Acculturative Stress Scale [11] precisely because, as Rodriguez and colleagues argue, it is not an inherent aspect of acculturation.

According to some proponents the effects of perceived discrimination and low socioeconomic status (SES), which often co-occur with acculturation, have been confounded with and misattributed to acculturation processes [12, 13]. In addition, research shows that socioeconomic and other general psychosocial factors are central to explaining much of the variance found in the mental health of migrants [14, 15]. Thus, it appears that acculturative stress may be better predicted by these risk factors.

### Measurement Issues and Tools on Immigration-Related Stress

To our understanding immigration-related stress is a broader concept than acculturative stress. There are many stressors immigrants may face at host countries, other than the potential cultural changes at individual and community levels. These stressors may include socioeconomic challenges, work-related difficulties and harsh living conditions. The main body of research on migration and stress focus on particular culturally diverse migrant groups such as Mexican migrant workers [13, 16], foreign students [17–20], elderly Arabs [21], Latino adolescent drug users [22], Southeast Asian refugees [23], and Indian migrants [24]. Indeed, the bulk of studies focus on one target population, to the extent that several instruments have been developed with one particular population group in mind. Examples of this are for instance the Hispanic Stress Inventory [25–27]; or the Multidimensional Acculturative Stress Inventory [11] both developed to assess acculturative stress among persons of Latin-American origin living in the United States.

In contrast, the Demands of Immigration Scale [28] focuses on migration-related issues for migrants preceding from any cultural origin. The scale consists of 23 items divided into 6 subscales: Loss, Novelty, Occupation, Language, Discrimination, and Not at Home. It shows good psychometric properties and has been validated in a number of different languages, including Arabic [29] and Chinese [30], and has been used with other migrant groups such as Latinos in the United States [31].

While the Demands of Immigration Scale is a promising assessment instrument, the Spanish and European immigration context, coupled with research that emphasizes the significance of various general psychosocial stressors, calls for a more sensitive tool that can capture the heterogeneity of the immigrant population in a specific local context. To that end, the Barcelona Immigration Stress Scale was developed [10]. The instrument consisted of 42 items divided into 4 subscales: Rejection, Homesickness, Hopelessness and Discrimination.

### Emic vs. Etic Approaches

As noted, many of the existing instruments associated with immigration stress have been developed for use with specific cultural groups. This clearly has the advantage of responding to the specific situation and characteristics of the different migrant groups, consistent with what has been called “cultural psychology” perspective which is “emic” in its focus on specific cultural groups [32]. This approach makes sense in that it allows for the specificity of the construct to each group; however, this approach lacks the “cross-cultural psychology” or “etic” approach which allows for cross-cultural comparisons and understanding [33]. The difficulty with cross-cultural comparisons, however, relies on the fact that cultural differences are such that certain constructs are derived from such different cultures that it would make no sense to treat them as belonging to the same entity.

The question of whether to adopt an emic or etic approach to mental health is a complex issue in the measurement field. “Equivalence” [34] is the implicit notion that exists in any intergroup study in which it is assumed that variance in a given construct is a function of real variance rather than group membership [35]. At the same time, research increasingly indicates the centrality of culture in most everything human as exemplified by the cultural neurosciences [36]. The meaning of any given notion or experience will have its particular cultural nuances, even within a given culture. Moreover, research as such would be extraordinarily limited if it strictly follows an emic or relativist approach, in which any given construct can only be examined within a particular cultural context, rendering intercultural or comparative research impossible [37, 38].

### The Barcelona Immigration Stress Scale (BISS)

The BISS was intended as a self-administered instrument capable of agilely measuring stress levels related to the migration process. It was designed to be suitable for use in both epidemiological and clinical studies. The first psychometric evaluation of the BISS was completed with a small number of immigrants in the Barcelona region [10]. It is noteworthy that no Latin Americans were included in this initial sample. Although the initial validation was carried out with the general “immigrant group” taken as coherent, both conceptually and driven by research on similar datasets, we consider it important to acknowledge heterogeneity and as such not simply assume that different migrant groups will perform in the same way. The cultural competence aspects of such instruments pertain to their flexibility in screening across domains that are adjusted to the situational needs of migrants. Although nominally consistent with the proposed model, the original factor structure did not fit satisfactorily. In fact, the authors concluded that the instrument was best suited for measuring a single construct.

The objective of this study is to reexamine the psychometric properties of the BISS with immigrants residing in Catalonia, Spain. Specifically, the study will examine the internal validity of the instrument following both etic and emic approaches. Our aim is to effectively capture the diverse experiences of acculturative stress by utilizing the BISS as a culturally competent instrument.

## Method

### Previous Scale Development

As commented in the introduction, an initial set of 42 items was developed and validated by Tomás-Sábado and colleagues [10] including subscales on Rejection, Homesickness, Hopelessness and Discrimination. This preliminary version of the scale showed a strong one-dimensional structure with a very high reliability close to one (α = 0.94). Regarding the anchor points, a four-point Likert scale (totally agree, moderately agree, moderately disagree, totally disagree) was chosen. This mode allows to avoid middle response bias [39]. The scale has been used in several studies on acculturative stress [e.g. 40, 41].

### Sample and Procedure

Participants of this study were gathered from a large primary care study comparing mental health, substance use and somatization among immigrants with their native counterparts [42, 43]. A total of 20 primary care centers from the greater autonomous region of Catalonia, most of them located in the greater metropolitan area of Barcelona, participated in this study.

The sample used for the further psychometric validation of the Barcelona Immigration Stress Scale (BISS) comprised a total of 915 immigrants residing in the Barcelona province. Among these, there were North Africans (14.3%), Eastern Europeans (7.2%), Sub Saharans (12.8%), Latin Americans (47.2%) and Asians (18.5%). The sampling strategy was based on the ethnic composition of foreign individuals utilizing primary care services. Because of language familiarity, there was a certain bias towards those participants who could respond to the questionnaires without comprehension barriers.

The average age was 33.25 (± 8.9), with a range from 17 to 67 years. Regarding sex composition, 60.7% of the sample were women. The average time since migration to Spain was five years (± 4.2) with a range of 1 month to 30 years. 25% of the sample had completed higher education. In addition, a quarter of the sample was currently unemployed. Three quarters of the sample reported a monthly income between one and two times the Spanish National Minimum Wage. Relatedly, three quarters of the sample had a working permit and 82% a residence permit.

The study received ethical clearance from the University Hospital Vall d’Hebron Institutional Review Board. All participants gave informed consent, and the data collection was completed anonymously.

### Analysis

Before analyzing the data, we carried an analysis of extreme cases by calculating the mean of all responses for each participant.

To decide upon its inclusion in principal component and confirmatory factor analyses (PCAs and CFAs) we calculated frequencies, skewness, kurtosis, and Item Response Theory (IRT) discrimination parameters for each item. Skewness measures the degree of asymmetry and Kurtosis measures the degree of peakedness of a distribution. The IRT discrimination parameter represents the degree to which the item discriminates between individuals who have high levels and those who have low levels of the trait being measured. We considered adequate (symmetric, mesokurtic and discriminant) those items with skewness and kurtosis values between 1 and − 1 and IRT discrimination parameters above 0.5.

Once we removed items that were considered asymmetric, leptokurtic, platykurtic, or non-discriminant, we calculated Cronbach’s alpha for the remaining set of items, as well as for each individual item if deleted. We then used PCA, a data-driven technique, to identify underlying dimensions within our set of observed variables (items). Through consecutive PCAs we aimed at reducing the number of items and identifying stable and theoretically congruent structures that conform the underlying structure of the scale. CFA, on the other hand, was used as a model-driven technique to tests the underlying dimensions obtained through PCA. To prevent overfitting [44], a cross-validation was performed by randomly dividing the sample into two parts, with one undergoing PCA and the other undergoing PCA. Finally, Cronbach’s alpha was used to test the internal consistency of all model steps and final subscales in the entire sample, as well as by ethnicity. All these analyses were performed first for the whole ethnically diverse sample, and, once a coherent, well-fitted structure was identified, for each of the ethnic groups. The psych [45], lavaan [46], and ltm [47] packages for the R software were used to compute all the statistical analyses.

## Results

Following an extreme cases analysis, we excluded 31 questionnaires with extreme response patterns or incomplete data. As a result, the final database for analysis consisted of 884 participants.

### Calculations with No Ethnic Group Differentiation

Frequencies, skewness, kurtosis and IRT discrimination parameters for each item are displayed in Table 1. As discrimination was suitable for all items, items that had skewness and kurtosis greater than 1 or less than − 1 were removed (7 items, see Table 1). We then calculated alpha for the whole scale and alpha if item deleted for each item, finding good values (*α* = 0.922 for the 35 items remaining, all items above *α* = 0.919).

**Table 1:** Descriptive, skewness, kurtosis and IRT parameters of the initial 42-item pool

Item	Title	Frequencies (%)				Skewness	Kurtosis	IRT discrimination
		Totally agree	Moderately agree	Moderately disagree	Totally disagree			
**1	I do not feel good in this country.	8.3	11.5	8.9	71.3	− 1.468	0.667	2.719
*2	Since I moved here, my life has gotten worse.	5.6	7.3	11.1	76.0	− 1.954	2.638	2.665
*3	I often feel that I am treated like a delinquent.	3.6	5.0	4.7	86.7	− 2.834	6.964	2.592
4	People here think that immigrants do not have the same social rights.	28	18	12.7	41.2	− 0.193	− 1.641	2.640
5	I regret having left my country.	12,4	10,3	11,4	65,8	− 1.227	− 0.065	3.558
6	People here would never accept an immigrant in their family.	10,2	11,2	16,1	62,5	− 1.217	0.073	3.095
**7	I cannot handle the pace of life in this country.	10,0	9,4	10,9	69,6	− 1.442	0.581	3.323
8	I am not sure if I want to stay here.	30,0	11,1	10,0	48,9	− 0.363	− 1.657	1.436
9	I feel guilty for having left my family.	17,4	9,9	6,7	65,9	− 1.041	− 0.662	3.328
10	Society constantly reminds you that you are an immigrant.	32,8	12,2	9,8	45,1	− 0.218	− 1.74	2.983
11	In this country, immigrants do not have the opportunity to obtain higher− ranking jobs.	26,5	12,3	11,6	49,5	− 0.453	− 1.537	2.595
12	To succeed here one has to renounce one’s culture.	15,1	6,6	7,8	70,6	− 1.335	0.066	3.663
13	I feel that people often do not include me in their activities because I belong to a different culture.	14,5	7,1	8,6	69,8	− 1.325	0.069	5.047
14	It bothers me that people here do not understand my cultural values.	16,4	8,5	10,2	64,8	− 1.107	− 0.459	4.376
*15	I do not feel accepted here.	8,1	9,1	11,3	71,6	− 1.594	1.142	4.035
16	People do not trust me because I am an immigrant.	17,6	10,5	12,3	59,6	− 0.922	− 0.811	4.907
*17	I am treated worse because of my appearance.	8,8	6,5	8,8	75,9	− 1.807	1.79	4.672
18	I feel discriminated against when it comes to finding housing.	18,7	7,4	8,4	65,4	− 1.055	− 0.643	3.842
19	I feel pressured by the people of this country to adopt their lifestyle.	11,6	8,0	9,7	70,7	− 1.453	0.535	3.980
20	I feel observed when I enter a store because they suspect that I will steal something.	20,3	9,1	7,3	63,3	− 0.914	− 0.942	3.303
21	I feel alone.	25,9	11,1	7,0	55,9	− 0.571	− 1.48	3.368
22	I cannot put up with the situation, which I am in for much longer.	11,7	9,2	8,6	70,6	− 1.397	0.349	4.651
23	I am worried that I cannot support my family.	35,8	11,0	6,3	46,9	− 0.169	− 1.815	2.504
24	I frequently feel tense.	24,2	14,7	10,6	50,4	− 0.475	− 1.495	3.629
25	I have financial problems.	29,2	17,4	14,4	39,0	− 0.153	− 1.642	2.210
26	I am very worried about my health.	42,7	12,9	9,5	34,8	0.193	− 1.747	1.460
27	I feel very bad when I think about everything I left behind in my country.	32,5	13,8	10,8	42,9	− 0.170	− 1.731	3.974
28	I feel that people observe me when I am out in public.	14,7	10,4	6,7	68,1	− 1.170	− 0.337	4.063
*29	I feel that I have failed.	9,1	5,0	7,6	78,3	− 1.943	2.254	4.351
30	It is very difficult for me to solve my problems.	18,8	11,3	10,7	59,3	− 0.851	− 0.973	4.316
*31	It worries me that I have involved other people in my decision to immigrate.	12,1	7,5	4,1	76,2	− 1.571	0.75	4.546
32	I have too many responsibilities.	42,3	12,5	9,3	35,9	0.161	− 1.767	2.247
33	I do not have adequate housing.	22,0	7,5	8,8	61,7	− 0.869	− 1.032	1.900
34	I feel like I have abandoned my family.	20,3	8,3	6,6	64,9	− 0.961	− 0.872	4.183
35	I do not trust the people of this country.	11,3	8,4	9,9	70,5	− 1.446	0.527	3.469
36	I miss my family.	66,9	12,9	5,4	14,8	1.321	0.126	1.628
**37	It worries me that I cannot educate my children according to my culture.	21,9	9,1	6,2	62,9	− 0.849	− 1.087	1.926
38	I miss the ambience of my hometown.	55,4	15,7	7,1	21,9	0.805	− 1.047	1.499
* 39	It is difficult for me to practice my religion.	9,9	4,2	3,3	82,6	− 2.070	2.582	3.825
**40	I feel that I will fail in this country.	16,7	7,3	6,4	69,6	− 1.215	− 0.286	4.153
41	I have felt that my culture is undervalued.	11,7	8,1	8,6	71,6	− 1.461	0.53	4.235
**42	I feel that I do not belong to this society.	19,6	11,7	9,4	59,4	− 0.814	− 1.06	4.664

*Removed for being asymmetric and or lepto/platykurtic. **Removed within factorial calculations

Consecutive principal component analyses using Varimax rotations were carried with the 35 remaining items using the first random half of the database. We fist used the eigenvalue higher than one criterion and then forced the structure to 3 and 4 factors. This procedure was repeated, excluding five items with low (< 0.4) and/or distributed loadings. Therefore, we identified a coherent 30-items three-factor structure. There were small differences between three and four factor-solutions in terms of variance explained (46.6–42.3%). Additionally, when we constrained to a four-factor solution, the items from the two factors that explained less variance were combined into a single factor. Therefore, we decided to proceed with a three-factor solution (Table 2). The dimensions of the model were named as follows: Discrimination (variance explained 29.6%, items 4, 6, 10, 11, 12, 13, 14, 16, 18, 19, 20, 28, 35, 41), Psychosocial Stress (variance explained 7.3%, items 21, 22, 23, 24, 25, 26, 30, 32, 33) and Homesickness (variance explained 5.4%, items 5, 8, 9, 27, 34, 36, 38).

**Table 2:** Principal component analysis of the selected 30 items with the ethnically diverse sample and stratified by ethnicity

ItemsN	Ethnically diverse*				North Africans				Eastern Europeans				Sub-Saharans				Latin Americans				Asians			
	D	S	H	IRT**	D	S	H	IRT*	D	S	H	IRT*	D	S	H	IRT*	D	S	H	IRT*	D	S	H	IRT*
4 People here think that immigrants do not have the same social rights.	0.597			2.268	0.579			1.519	0.760			2.250	0.683			1.612	0.611			1.711	0.602			1.614
5 I regret having left my country.			0.571	2.688	0.441	0.585		1.646	0.588	0.235		1.148	0.676	0.125		0.698		0.321	0.575	1.902	0.519	0.244		1.361
6 People here would never accept an immigrant in their family.	0.521			2.708	0.564	0.266		1.747	0.598			1.267	0.574		0.264	1.791	0.450			1.178	0.566			1.515
8 I am not sure if I want to stay here.			0.524	1.383		0.434		0.830		0.366		1.166	0.398	0.196	0.163	0.855	0.286		0.531	0.931			0.401	0.427
9 I feel guilty for having left my family.			0.613	2.507		0.693		1.369		0.587		1.636	0.262	0.499	0.268	3.162	0.210	0.330	0.523	1.920			0.644	1.432
10 Society constantly reminds you that you are an immigrant.	0.629			2.730	0.553	0.234	0.217	2.537	0.649			1.623	0.626	0.190		1.764	0.663			1.807	0.601			1.047
11 In this country, immigrants do not have the opportunity to obtain higher-ranking jobs.	0.503			2.264	0.609			2.253	0.393	0.575	0.262	2.011	0.580		0.245	1.517	0.476	0.245		1.181	0.655			1.461
12 To succeed here one has to renounce one’s culture.	0.606			3.631	0.608	0.219		2.159		− 0.314	0.499	0.597	0.455	0.184		1.303	0.592			1.611	0.663			1.666
13 I feel that people often do not include me in their activities because I belong to a different culture.	0.642			5.845	0.709	0.318		4.709	0.706			3.584	0.633		0.211	2.080	0.619	0.248		2.085	0.584			1.486
14 It bothers me that people here do not understand my cultural values.	0.677			4.283	0.613	0.329		3.000	0.667			1.693	0.688			2.171	0.651		0.232	2.090	0.580		0.342	1.587
16 People do not trust me because I am an immigrant.	0.663			4.151	0.574	0.361		2.837	0.716		0.300	4.349	0.647	0.194		1.448	0.641		0.235	2.354	0.440	0.308		1.235
18 I feel discriminated against when it comes to finding housing.	0.533			2.817	0.624			1.906			0.250	0.897	0.483		0.286	1.275	0.569	0.217		1.598	0.288	0.208	0.310	1.104
19 I feel pressured by the people of this country to adopt their lifestyle.	0.567			3.019	0.572	0.279		2.487	0.333			1.066	0.484		0.257	1.521	0.547	0.273		1.655	0.290		0.489	0.999
20 I feel observed when I enter a store because they suspect that I will steal something.	0.565			2.669	0.527		0.424	1.860	0.497	0.307	0.327	1.946	0.519		0.298	1.193	0.535	0.201		1.554	0.403	0.376		1.158
21 I feel alone.		0.462		2.523		0.256	0.472	1.344			0.690	1.192	0.230	0.248	0.629	1.783	0.228	0.475	0.378	1.611	0.236	0.542		1.222
22 I cannot put up with the situation, which I am in for much longer.		0.413		3.001	0.525		0.352	1.292			0.787	1.855	0.140	0.116	0.494	1.035	0.341	0.534	0.223	2.014	0.422	0.331	0.340	1.904
23 I am worried that I cannot support my family.		0.730		3.459			0.612	1.357	0.234	0.496	0.318	1.888		0.585	0.224	1.264		0.680		1.680		0.517	0.238	1.104
24 I frequently feel tense.		0.573		4.518	0.225		0.697	1.921	0.301	0.280	0.628	2.530	0.263	0.233	0.609	1.916	0.324	0.611		1.965	0.285	0.595		1.649
25 I have financial problems.		0.747		3.589	0.310		0.605	1.621		0.444	0.319	1.274	0.116	0.532	0.403	1.896		0.710		1.780		0.748		1.117
26 I am very worried about my health.		0.500		2.008		0.252	0.590	1.003		0.534		0.689	− 0.122	0.393	0.211	0.760		0.502		1.114		0.606	0.275	1.061
27 I feel very bad when I think about everything I left behind in my country.			0.646	4.717		0.734	0.242	2.897		0.539	0.220	2.923	0.433	0.402	0.320	2.280		0.431	0.650	2.861	0.318	0.442	0.350	1.757
28 I feel that people observe me when I am out in public.	0.569			3.036	0.601			1.767	0.411	0.507	0.251	1.902	0.616			1.438	0.531	0.287		1.809	0.407	0.328	0.254	1.125
30 It is very difficult for me to solve my problems.		0.641		5.581			0.715	1.544		0.499	0.541	2.434	0.198	0.368	0.500	1.820	0.294	0.685		3.157	0.316	0.460	0.323	1.779
32 I have too many responsibilities.		0.560		2.304		0.251	0.406	0.868	0.255	0.289		0.796		0.581		0.688	0.232	0.580		1.330		0.625		1.166
33 I do not have adequate housing.		0.451		2.230			0.331	0.685		0.514		0.781			0.679	0.852	0.234	0.505		1.358	0.306		0.291	0.769
34 I feel like I have abandoned my family.			0.623	3.394		0.644		1.687		0.495		1.184	0.224	0.548	0.211	2.207		0.414	0.587	2.744		0.380	0.471	1.649
35 I do not trust the people of this country.	0.528			3.052	0.398	0.463		1.889	0.566			1.456	0.511	0.279	− 0.327	0.945	0.534	0.254		1.845	0.411		0.337	1.082
36 I miss my family.			0.599	1.727		0.527	0.355	1.867		0.676		0.748		0.619		0.709			0.658	1.513			0.585	1.074
38 I miss the ambience of my hometown.			0.606	1.785		0.535	0.264	1.393	0.251	0.501	− 0.214	0.764	0.187	0.498		0.683			0.678	1.466			0.618	0.909
41 I have felt that my culture is undervalued.	0.620			2.867	0.511		0.343	1.751	0.645			1.930	0.557	0.106		1.203	0.590			1.638	0.264	0.357		1.018

*Performed using the first random half of the database, **Calculated within each subscale using the complete database

*D* Discrimination, *S* Psychosocial stress, *H* Homesickness

**Fig. 1:**
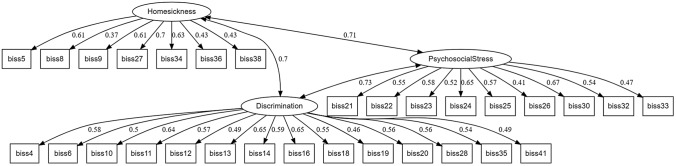
Path diagram summarizing the multidimensional fit of the final confirmatory factor analysis (30 items). *The values displayed on the arrows represent standardized factor loadings and collinearity between factors. These parameters indicate the strength and direction of the relationship between each latent construct and its observed indicators or between them

We assessed the unidimensional and multidimensional fit of the model through confirmatory factor analyses, using the second random half of the database. Table 3 shows unidimensional and multidimensional fits for the initial 35-item structure, the final 30-item structure, as well as unidimensional fit parameters for each final subscale. Figure 1 shows the CFA path diagram of the final model. All models demonstrated an acceptable fit, and given the negligible differences, the reduction from 35 to 30 items was deemed justifiable.

**Table 3:** Reliability and confirmatory factor analysis fit parameters

	MTBM	CFI	TLI	RMSEA	SRMR	Alpha
Ethnically diverse migrant sample						
Unidimensional fit after excluding asymmetric and or lepto/platicurtic items (35 items)	4262.33	0.798	0.786	0.059	0.059	0.922
Multidimensional fit (35 items)	4262.33	0.870	0.861	0.047	0.052	
Unidimensional fit (final 30 items)	3657.79	0.788	0.772	0.065	0.062	0.911
Multidimensional fit (30 items)	3657.79	0.882	0.872	0.049	0.051	
Final subscales’ unidimensional fit						
Discrimination (14 items)	1545.33	0.911	0.894	0.064	0.049	0.872
Psychosocial stress (9 items)	795.18	0.948	0.930	0.059	0.042	0.801
Homesickness (7 items)	549.20	0.923	0.884	0.083	0.051	0.754
Multidimensional analyses by ethnicity						
North Africans	1412.94	0.764	0.745	0.073	0.088	0.897
Eastern Europeans	1066.77	0.503	0.462	0.116	0.124	0.862
Sub-Saharans	1198.25	0.753	0.732	0.071	0.085	0.887
Latin Americans	4507.38	0.886	0.877	0.053	0.050	0.921
Asians	1422.61	0.751	0.730	0.067	0.076	0.892

*MTBM* Model Test Baseline Model, *CFI* Comparative Fix Index, *TLI* Tucker Lewis Index, *RMSEA* Root Mean Square Error of Approximation, *SRMR* Standardized Root Mean Square Residual

### Calculations by Ethnic Group Differentiation

Tables 2 and 3 offer factor loadings and fits of the previously validated model by ethnic group. Although fits and internal consistency were deemed satisfactory for all ethnic groups, the Latin American group demonstrated a better fit compared to the other groups. Conversely, the Eastern European group exhibited the worst fit parameters.

## Discussion

We have validated a shortened version of the Barcelona Immigration Stress Scale, consisting of 30 items, which exhibits improved psychometric properties. This new validation has been developed both considering the overall group of migrants and differentiating ethnic origins. The final structure of the BISS demonstrated an adequate fit according to IRT, PCA, and CFA parameters and good internal consistency. The three subscales found (Discrimination, Psychosocial Stress, and Homesickness) coincide approximately with the structure and variance explained by the subscales of the original validation (Rejection, Discrimination, Hopelessness, and Homesickness; [10]). According to our findings, we consider that the further validation of the BISS has added value to the instrument in a way that best reflects the common areas of distress experienced by migrants, who were attending primary health care centers in Catalonia, Spain. The sample for its further validation (*n* = 915) was larger and more representative to the immigrant population in the region, than the one (*n* = 92) examined for its preliminary validation [10]. Moreover, the further validated version of the scale emphasizes the need to assess migration stress across multiple domains that are tailored to the specific situational needs of the migrant.

Results show three final dimensions/subscales, namely: Discrimination, Psychosocial Stress and Homesickness. The Discrimination subscale addresses the perceived discrimination on migrants’ acculturation [52, 53]. Perceived discrimination can be conceived as a belief that one has been treated unfairly because of one’s origin. It may result from a sense of being differentially treated in public places or of being barred access to sources of information, social networks, and peer groups. In a sense, perceived discrimination indicates the nature of the interaction between migrants and the receiving society. It shows the incongruence between the orientation and expectations that migrants and the receiving society have of each other [54]. It constitutes a negative life experience and a potential source of chronic stress and, thus, can explain deficiencies in migrants’ well-being and health, resulting in a disruptive effect on the social adjustment in the host society [55].

Psychosocial Stress within our instrument refers to any everyday life stressors that may condition the wellbeing of the migrant, such as issues related to housing, family, work and other responsibilities [56, 57]. Such stressors are not unique to the post-migration phase since native populations may also face similar life challenges in everyday life in the same social contexts where migrants are studied.

Homesickness refers to the distress caused by actual or anticipated separation from familiar or loved people or places. According to Thurber [58], it is mostly accompanied by cognitive components such as acute longing and intrusive thoughts about home and attachment objects. Stroebe and colleagues [59] have proposed that homesickness results from the combined effects of loss (loss-orientation) and adjustment to the new situation (restoration-orientation). Just as grieving people must cope with the loss experience and changes to their circumstances, homesick individuals must cope with the loss (even if temporary) of their family and friends, as well as their changed circumstances.

As commented above, the psychometric properties of the scale, regardless of origin, were very good, both in relation to the discriminative power of the items, internal consistency, and fit. Upon conducting analyses by ethnic group, we found that the Latin American sample, which was the largest, exhibited the most favorable reliability and fit parameters. Conversely, the other ethnic groups displayed poorer reliability and fit parameters, with particularly lower fit observed among individuals from Eastern Europe. Although the discriminatory power of specific items was generally adequate for all ethnic groups, some illustrative examples within Eastern European participants can be seen. For instance 12 “To succeed here one has to renounce one’s culture” (0.597), 18 “I feel discriminated against when it comes to finding housing” (0.897), 26 “I am very worried about my health” (0.689), 32 “I have too many responsibilities” (0.796), 33 “I do not have adequate housing” (0.781), 36 “I miss my family” (0.748) and 38 “I miss the ambience of my hometown” (0.764). Some of these items had also low discriminant power in the case of North Africans (32, 33) and Sub-Saharans and Asians (26, 32, 33, 36 and 38) and additionally, 8 “I am not sure if I want to stay here” (0.830 for North Africans and 0.427 for Asians).

Eastern European immigrants in Barcelona hold indeed some particularities in relation to the rest of the non-EU immigrants in the city. Social relations between Eastern Europeans and the native population are often described in terms of cultural proximity (e.g., religion) and high levels of integration in the Spanish society. However, interaction between Eastern European migrants and other migrant groups are described in terms of distance and prejudiced views influenced by negative rhetoric about non-white and non-European migrants in Spain [48]. Ramírez Goicoechea [49] highlights the “invisibility” of Eastern European migrants in Spain because of their physical similarity of them to Catalans and Spaniards. As a result, Eastern Europeans in Spain may experience lower levels of acculturation stress, possibly due to encountering less discrimination and prejudice. Future community-based studies are needed to confirm this statement.

Discrimination appears to be a universal explanatory factor of migration stress for all immigrants, regardless of their ethnic origin, as it showed a very good fit in all groups. Immigrants may be more vulnerable to certain types of discrimination than the native born [50] and the very perception of discrimination is related to the perception of specific health symptoms, such as stress [51]. Our findings may imply that perceived discrimination may not be necessarily related to attitudes by the host community but by other migrant ethnic groups too, as this may apply to the Eastern European migrant community.

The clinical implications of the study include a culture-sensitive approach in screening for migration-related stress in primary health care. Migration stress may compromise the physical and mental health of individuals if not identified and addressed [2]. Early screening and detection of stressors and risk factors related to acculturation stress can contribute to the prevention of mental health problems in migrant populations [3]. Prolonged exposure to these stressors can have a destabilizing effect on mental health, and early detection is crucial to prevent further distress.

Among the limitations of the study, it is important to note that the sample we used may not be fully representative of migrants in Catalonia or in general. Our sample consisted of individuals who have access to primary care, which could have implications for the composition of the sample in terms of ethnicity, gender, socioeconomic status, and legal status. Regarding the latter, in the case of Spain, access to primary healthcare does not necessarily require legal residency, but there may be an overrepresentation of individuals with work and residence permits. Consequently, the method of sample selection employed in this study may have resulted in a scale with a different dimensional structure compared to what would have been obtained in a different context.

The present validation of the BISS adopts an etic approach while acknowledging that migration stress is a multi-dimensional construct, and its effective measurement in primary health care depends on the flexibility in understanding the situational needs of every individual across the three subscales identified independent of their ethnic origin. To establish its generalizability and applicability across diverse immigrant populations, the further validated scale would also require testing in primary health care settings outside Catalonia, as well as in other clinical settings such as mental health specialty units and in the community.

## Conclusions

In conclusion, the newly validated BISS scale has demonstrated good psychometric properties and specificity in the structure of the model. As a multidimensional instrument, it can help to understand the various sociocultural factors that may cause distress to migrants, especially in primary healthcare settings. Further testing of the scale in different clinical and community settings outside Catalonia may provide more evidence for its utility in the assessment and prevention of mental health problems related to migration. In this regard, early detection and screening of migration stress and risk factors using the BISS can contribute to the prevention of mental health problems among migrants.

### Supplementary Information

Below is the link to the electronic supplementary material.

Supplementary file1 (PDF 162 KB)

Supplementary file2 (PDF 163 KB)

Supplementary file3 (CSV 147 KB)

Supplementary file4 (TXT 11 KB)
