# Supplementary material for: Cross-Cultural Validation of the Barcelona Immigration Stress Scale
Source: J Immigr Minor Health. 2023 Jul 17;25(6):1339–53. doi: 10.1007/s10903-023-01520-2 (PMC10632226; doi:10.1007/s10903-023-01520-2)
Supplement: Supplementary file 1 — Supplementary file1 (PDF 162 KB) [file 10903_2023_1520_MOESM1_ESM.pdf]

Barcelona Immigration Stress Scale (BISS — 30)

	Strongly agree (1)	Moderately agree (2)	Moderately disagree (3)	Strongly disagree (4)
1 I do not have adequate housing.				
2 I feel that people often do not include me in their activities because I belong to a different culture.				
3 I feel that people observe me when I am out in public.				
4 I feel observed when I enter a store because they suspect that I will steal something.				
5 In this country, immigrants do not have the opportunity to obtain higher-ranking jobs.				
6 It is very difficult for me to solve my problems.				
7 Society constantly reminds you that you are an immigrant.				
8 I have financial problems.				
9 I am not sure if I want to stay here.				
10 I feel guilty for having left my family.				
11 I cannot put up with the situation, which I am in for much longer.				
12 To succeed here you have to give up your culture				
13 I miss the ambience of my hometown.				
14 I frequently feel tense.				
15 I feel alone.				
16 People here believe that immigrants are not entitled to the same social rights.				
17 I feel pressured by the people of this country to adopt their lifestyle.				
18 I feel like I have abandoned my family.				
19 I regret having left my country.				
20 People do not trust me because I am an immigrant.				
21 I feel very bad when I think about everything I left behind in my country.				
22 I do not trust the people of this country.				
23 I have too many responsibilities.				
24 I miss my family.				
25 I have felt that my culture is undervalued.				
26 I am very worried about my health.				
27 People here would never accept an immigrant in their family.				
28 It bothers me that people here do not understand my cultural values.				
29 I am worried that I cannot support my family.				
30 I feel discriminated against when it comes to finding housing.				

Items by subscales: Discrimination (2, 3, 4, 5, 7, 12, 16, 17, 20, 22, 25, 27, 28, 30), Psychosocial Stress (1, 6, 8, 11, 14, 15, 23, 26, 29), Homesickness (9, 10, 13, 18, 19, 21, 24). We recommend averaging the items of each subscale so that all scores range from 1 to 4. This scale has been built considering a dimensional perspective of stress and, therefore, does not consider the use of cut-off points.

Eiroa-Orosa, F. J., Evangelidou, S., Qureshi, A., & Collazos, F. (2023). Cross-cultural validation of the Barcelona Immigration Stress Scale. *Journal of Immigrant and Minority Health*. https://doi.org/10.1007/s10903-023-01520-2
