# Supplementary material for: Cross-Cultural Validation of the Barcelona Immigration Stress Scale
Source: J Immigr Minor Health. 2023 Jul 17;25(6):1339–53. doi: 10.1007/s10903-023-01520-2 (PMC10632226; doi:10.1007/s10903-023-01520-2)
Supplement: Supplementary file 2 — Supplementary file2 (PDF 163 KB) [file 10903_2023_1520_MOESM2_ESM.pdf]

Escala Barcelona de Estrés del Inmigrante (BISS — 30)

	Totalmente de acuerdo (1)	Moderadamente de acuerdo (2)	Moderadamente en desacuerdo (3)	Totalmente en desacuerdo (4)
1 No tengo una vivienda adecuada				
2 Siento que la gente, a menudo, no me incluye en sus actividades por pertenecer a otra cultura				
3 Siento que la gente me observa cuando voy por la calle				
4 Me siento observado cuando entro en una tienda porque sospechan que voy a robar algo				
5 En este país, el inmigrante no tiene oportunidad de acceder a cargos de categoría				
6 Encuentro muchas dificultades para solucionar mis problemas				
7 La sociedad constantemente te recuerda que eres inmigrante				
8 Tengo dificultades económicas				
9 No estoy seguro de querer quedarme aquí				
10 Me siento culpable por haber dejado a mi familia				
11 No podré aguantar mucho tiempo en la situación en la que me encuentro				
12 Para tener éxito aquí hay que renunciar a tu cultura				
13 Echo de menos el ambiente de mi pueblo				
14 Con frecuencia me siento tenso				
15 Me siento solo				
16 La gente de aquí cree que los inmigrantes no tenemos derecho a los mismos derechos sociales				
17 Me siento presionado por la gente de este país a adoptar su modo de vida				
18 Siento que he abandonado a mi familia				
19 Me arrepiento de haber dejado mi país				
20 La gente desconfía de mí por ser inmigrante				
21 Me siento muy mal cuando pienso en todo lo que dejé en mi país				
22 Desconfío de la gente de este país				
23 Tengo demasiadas responsabilidades				
24 Echo de menos a mi familia				
25 He sentido que mi cultura era despreciada				
26 Me preocupa mucho mi salud				
27 La gente de aquí nunca aceptaría a un inmigrante en su familia				
28 Me siento molesto porque la gente de aquí no entiende mis valores culturales				
29 Me preocupa no poder mantener a mi familia				
30 Me siento discriminado a la hora de acceder a una vivienda				

Ítems por subescalas: Discriminación (2, 3, 4, 5, 7, 12, 16, 17, 20, 22, 25, 27, 28, 30), Estrés Psicosocial (1, 6, 8, 11, 14, 15, 23, 26, 29), Nostalgia (9, 10, 13, 18, 19, 21, 24). Recomendamos promediar los ítems de cada subescala para que todas las puntuaciones tengan un rango de 1 a 4. Esta escala se ha construido desde una perspectiva dimensional del estrés y, por lo tanto, no considera el uso de puntos de corte.

Eiroa-Orosa, F. J., Evangelidou, S., Qureshi, A., y Collazos, F. (2023). Cross-cultural validation of the Barcelona Immigration Stress Scale. *Journal of Immigrant and Minority Health*. https://doi.org/10.1007/s10903-023-01520-2
